# Biophilic smart cities: the role of nature and technology in enhancing urban resilience

**DOI:** 10.1186/s44147-021-00042-8

**Published:** 2021-12-05

**Authors:** Sara Tarek, Ahmed Salah El-Din Ouf

**Affiliations:** Dept. of Arch. Engineering, Faculty of Engineering, Cairo University, Cairo, Egypt

**Keywords:** Biophilic cities, Digital technology, Restorative urbanism, Smart cities, Urban resilience

## Abstract

The purpose of the study is to identify the main indicators and aspects for applying biophilic and smart solutions in city planning and design to achieve urban resilience. This research investigates the main definitions, elements, and attributes for biophilic design and smart city planning that help in developing resilience strategies for healthier urban environments. This study follows a methodology that comprises two approaches; first a concise review definition and principles of urban resilience. Then investigating the notions of biophilic and smart approaches to achieve healthier urban environments. Second, an analytical approach that proposes a framework for applying biophilic and smart city indicators to achieve urban resilience. The proposed framework highlights the achieved adaptive capacities for resilient cities due to adopting biophilic and smart solutions. The research results highlight a proposed relationship between principles of urban resilience, biophilic city indicators, and smart city indicators.

## Introduction

Resilient cities are the result of flexible and adaptive capacities that respond to disasters and sudden shocks caused by both natural and man-made factors. Moreover, it is important to respond to the raised calls regarding the rapid development of Information technology and more integration with the natural environment, which has been slightly neglected as a topic of concern [1]. For example, one of the major lessons learned from dealing with the COVID-19 crisis was to look a step back to nature with its therapeutic restorative and healing influences to mitigate the spread of the disease [2, 3]. In addition, it was our smarter and innovative technology solutions that provided us with the vaccine in a few months.

Focusing on both nature and smart technology provides the means to the current needs of our communities. However, the idea of merging between nature and technology is not relatively new. There are different cities that adopted ideas of integrating green natural environments with digital technological tools to enhance communities’ connection to nature and achieve a sustainable environment, like “Melbourne Urban Forest Visual,” an initiative integrating forests with ICT in Australia.

In this regard, this study aims to identify the main dimensions and indicators for achieving urban resilience through biophilic and smart cities’ solutions. Accordingly, the research answers two main questions; what role could biophilic and smart cities play in enhancing urban resilience practices? Will the adoption of biophilic and smart city approaches provide new solutions and strategies for contemporary urban areas?

The presented study starts with a systematic literature review which ends by a proposed framework for achieving urban resilience using biophilic and smart approaches. The research has two parts: the first part is a literature review for relevant papers and research articles that are indexed in Scopus and Web of Science databases. The initial literature review was performed between July 2020 till June 2021 using the keywords: “biophilic cities or urbanism,” “smart cities,” “resilient cities.” The reviewed papers were categorized according to the research’s main objectives to explore the necessary theoretical data for the presented research. Results of the literature review introduced biophilic and smart city approaches that might act as dimensions and principles of resilient urbanism.

The second part of the research synthesizes the key findings of the literature review using a descriptive-analytical method to investigate the cross-relationship between biophilic cities’ concept and smart city approaches on one side and urban resilience on the other side. It studies both biophilic and smart cities’ indicators’ contributions to urban resilience principles. The paper ends by proposing biophilic-smart adaptive capacities to achieve urban resilience through environmental, socio-cultural, economic, and governance/managerial actions.

## Literature review

Resilience is more about recovery from a certain danger or disaster. It could be defined as the capability to become accustomed to shocks, resist various changes and rebounds [4–6]. Cities become resilient when they can make it through risk, danger or crisis, by using their inner strength and resolve besides adopting building forms and infrastructure [7]. Adopting resilience approaches and principles in strategies of cities helps communities live with risk and danger [7–9]. There is a strong relationship between resilience and sustainability with its social, economic, and ecological aspects. Both concepts aim to enhance the future life of communities by following preventive standards for resource use in the light of the emerging crises [10]. Different studies investigated the ability to adopt holistic approaches for urban resilience by distinguishing sustainability dimensions and indicators mainly; economy, society, and environment [11–13].

Accordingly, there were different types of outputs and findings such as the European economic and social wellbeing in the industrial regions and metropolitan areas of capital cities [14, 15]. Another output was the positive relationship between the territorial outcome and regional resilience drivers. which focused more on the economic factors in terms of innovation and investment in human capital [16, 17].

### Methodology of the review

The presented research follows a systematic literature review; the latest research in the field was collected from international journals and books to build an epistemic framework for combining biophilic and smart cities’ indicators to achieve urban resilience. Therefore, the review started by using the following keywords “biophilic cities,” “biophilic urbanism,” “smart cities,” and “resilient cities” in Web of Science and Scopus databases from 2013 to 2021. A total of 707 researches—including books, journal articles and conference proceedings—were found, which was considered reliable. To narrow down the selection only English academic journals and papers that are relevant to the research objective were selected. Accordingly, 237 journal articles in Web of Science and 304 journal articles in Scopus were used in the presented review and used to build the proposed framework.

### Definition and principles of urban resilience

The notion of resilience includes the idea of adaptive capacity which is an essential requirement for cities’ resilience [18] which is more about recovery from a certain danger or disaste r[4, 5]; it is the ability to plan, facilitate and implement the adaptation options [18]. Hence, it is important to include different dimensions for urban design while developing a framework for urban resilience such as; physical, environmental, economic, social, infrastructural, and institutional dimensions [18, 19]. Previous studies identified three forms of urban resilience, namely resistance, recovery, and transformation. Each one of the previous forms comes in different timing, before, during, and after the crisis respectively [19, 20]. Cities’ strategies should be implemented in different ways to ensure achieving resilience through embracing new opportunities raised from the social and environmental transformations that occurred as consequences of the current crisis, such as climate change and digital transformation [21, 22]. According to the previous literature principles of resilience can be divided into five main principles, which are fundamental to achieve urban resilience. Table 1 identifies these principles in addition to how they could be achieved according to previous studies [19, 20, 23], each of the five principles was given a code (R01–R05) to facilitate using it in the proposed framework.

**Table 1:** Principles of urban resilience adopted from [19, 20, 23]

Code	Principles	Recommendations
**R01**	Resources and materials availability, quality, and accessibility	Monitoring and protecting of the ecosystem. Reducing the environmental impact. Conserving wildlife and biodiversity. Managing materials and resources.
**R02**	Communities’ safety, culture, and wellbeing	Composing population. Connecting people using community groups. Sharing assets. Encouraging place attachment. Preventing and reducing crime. Providing security services. Enhancing physical and psychological health. Providing health measures. Learning from past experience dealing with disasters
**R03**	Economic structure’s security, stability, and dynamism	Increasing employment rates. Reducing illiteracy and increasing individual skills. Enhancing collective ownership of community assets. Increasing diverse economic structures and livelihood strategies. Encouraging private investment in addition to public - private ownership. Making balance in the local market in terms of supply and demand
**R04**	Built environment and infrastructure robustness and redundancy	Enhancing redundancy and robustness of infrastructures, buildings and facilities. Enhancing spatial distribution of critical infrastructure. Enhancing multi-functionality of spaces and facilities. Retrofitting and renewing of built environment. Establishing diverse, reliable ICT networks. Encouraging mixed use development and compact urban form. Developing diverse public spaces and communal facilities. Enhancing connectivity and streets' typologies. Enhancing green and blue infrastructures.
**R05**	Institutions and governance planning mechanisms and its effectiveness of community organizations relationships	Sharing integrated and updated long-term vision. Adopting multi-stakeholders planning and decision making. Decentralizing responsibilities and resources. Managing resources efficiently. Adopting professional emergency response and recovery skills. Integrating risk reduction into development policies and plans. Updating risk assessment scenarios for different infrastructures and organizations. Developing a standardized and updated data base for action planning, monitoring and evaluation. Including transit population in emergency planning. Adopting innovative technologies.

### Biophilic city patterns and indicators

Biophilic urbanism aims at connecting the urban setting to nature, integrating natural experience in contemporary modern urbanism, and solving ecological and social problems [24–28]. It deals with people as an essential part of the ecosystem. Also, it targets satisfactory experience for all people at different scales [29, 30]. Adopting biophilic elements in architecture design and urbanism is believed to have a direct impact on people’s health, like; reducing stress, emotional well-being, boosting creativity, healing effects, increasing productivit y[31–33]. Thus, such elements will make cities more resilient on the ecological, economic, and social levels [34, 35]. Previous studies adopted many methods to develop different lists for the elements of biophilic cities [25, 36]. However, most of the studies were more concerned with elements of biophilia on the architecture scale (interior and exterior); and few studies were more concerned with elements of biophilia on the urban scale [37, 38]. This study reviewed different biophilic design principles and patterns as summarized in Table 2.

**Table 2:** Biophilic principles and patterns adopted from [28, 39, 40]

Principles & patterns	Sub-principles (How to achieve it?)
Nature in the Space Patterns	Visual Connection with Nature; Non-Visual- Connection with Nature; Non-Rhythmic Sensory Stimuli; Thermal & Airflow - Variability, Presence of Water, Dynamic & Diffuse Light, Connection with Natural Systems
Natural Analogues Patterns	Biomorphic Forms & Patterns; Material Connection with Nature; Complexity & Order
Nature of the Space Patterns	Prospect; Refuge; Mystery; Risk/Peril.
Visual Connection with nature	Providing different views for natural urban elements.
Non-Visual connection with nature	Enhancing the other senses like hearing, smell and touch in experiencing the urban environment.
Presence of water	Improving the multi-sensory experience with water view and sound.
Connection with natural system	Using nature integration to urbanism in order to enhance ecological functions in the ecosystem.
Natural forms and patterns	Following organic and free forms to enhance biophilic design patterns and avoid using rigid straight forms.
Material connection with nature	Using natural materials to achieve more connection between people and nature.
Complexity	Using complexity in spatial design to enrich people’s experience and feelings; like what happens in experiencing nature.
Prospect	Making passable and smooth views while adopting biophilic design.

**Fig. 1:**
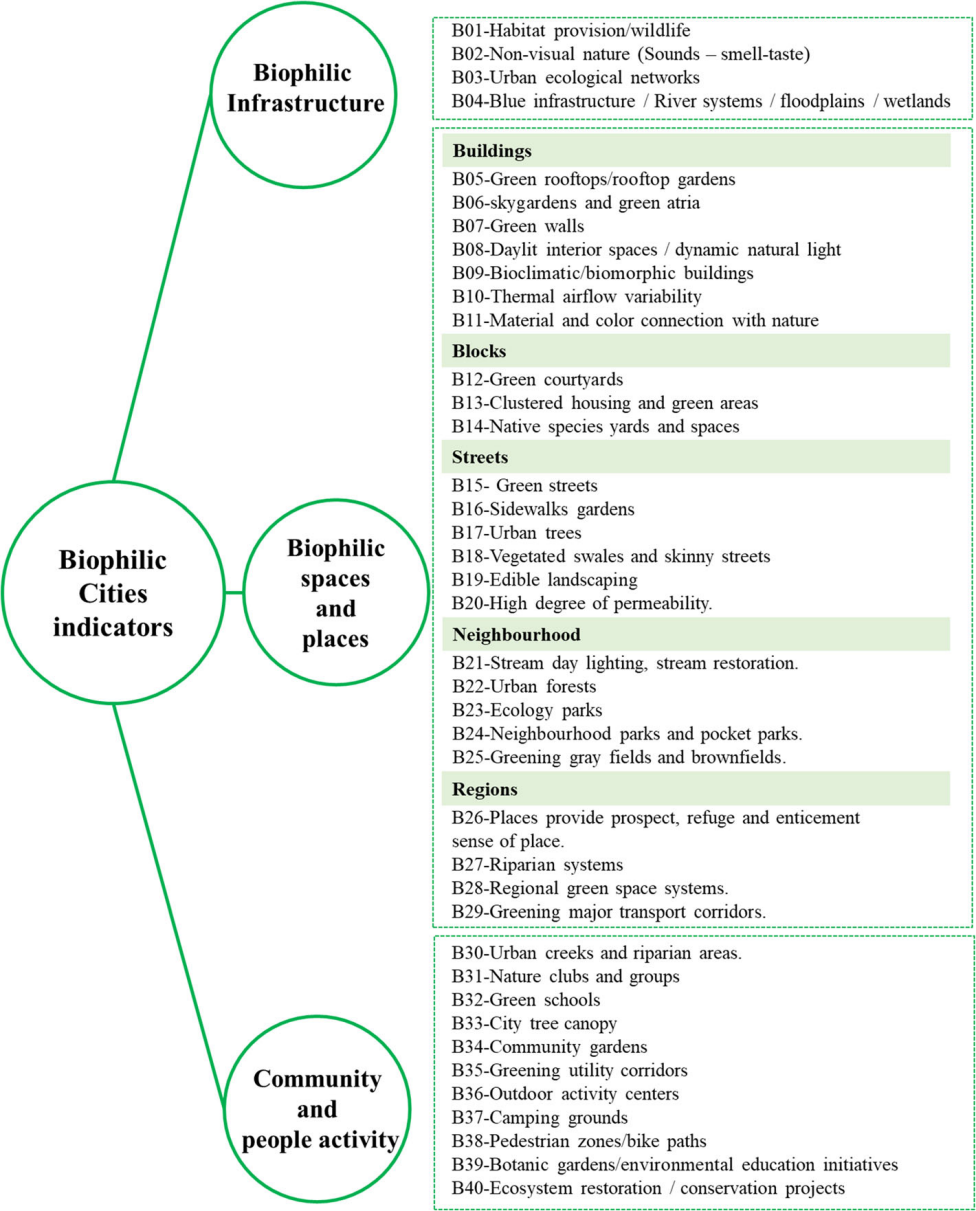
Biophilic city indicators adopted from [34, 35, 41–43]

Moreover, the study reviewed more than 50 indicators covering different urban scales and patterns of biophilic design. Only 40 indicators coded from B01 to B40 were selected to be adopted in this research study. The selected indicators are related to urban resilience, disasters, health, and covering urban level as shown in Fig. 1.

The indicators are presented into three main categories; Biophilic infrastructure, Biophilic spaces and places (buildings, blocks, streets, neighborhoods, and regions) in addition to community and people activity. The different categories are covering both physical and non-physical aspects so it was essential to select indicators that can be measured and applied in the proposed framework [31, 32, 39].

### Smart city initiatives, understandings, and indicators

The notion of a smart city could be considered a utopia. It comes from different interrelated studies in urbanism and Information and Communication Technologies (ICT) usage for development [44]. The complementary perspectives of smart cities comprise efficient technologically advanced, sustainable, and socially inclusive cities [45–47]. Many studies were conducted aiming to develop different sets of assessment indicators for smart cities [47–49]. Comprehensive outlines and overviews were conducted to achieve smartness in contemporary cities, attempting to develop frameworks for smart cities in addition to assessment tools and indicator sets [50–52]. The presented study reviewed well-established approaches and concepts for identifying smart city indicators and different initiatives for smart cities around the world. The reviewed studies and initiatives are; CITYKEYS’ indicators, Smart City PROFILES, City Protocol, Smart City Ranking and methods using the Key Performance Indicators (KPIs) and Triple-helix Network Model for Smart Cities Performance (SCP), Euro-pean Innovation Partnership on Smart Cities and Communities, ESETIS, Qatar smart program (TAS-MU), Smart Dubai, Smart Melbourne, Smart City Berlin Strategy, Urban Agenda for EU, City VITAlity and Sustainability (CIVITAS) [53–59].

**Fig. 2:**
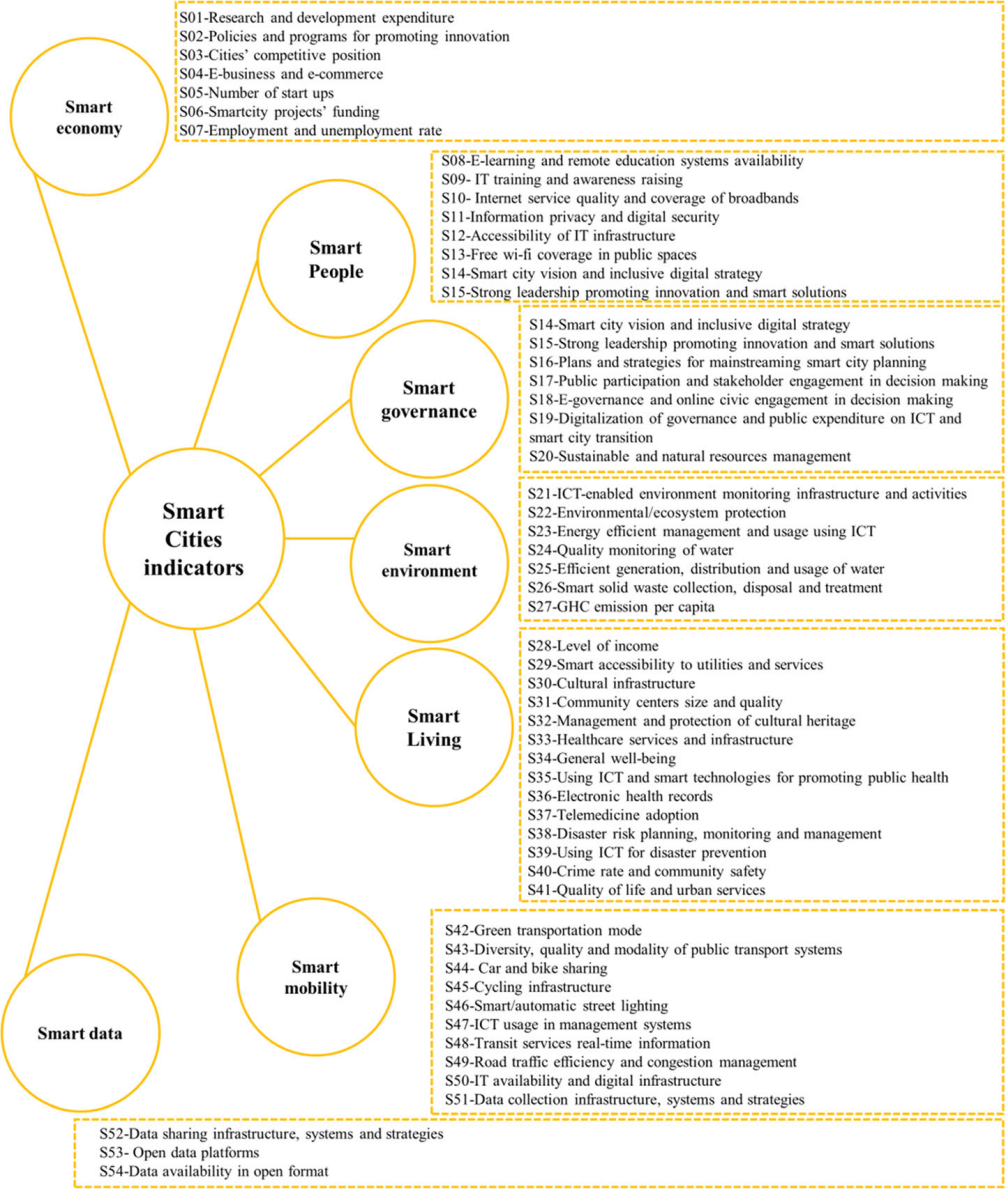
Smart city indicators adopted from [47, 48, 60–62]

This study adopted the European approach in categorizing the reviewed indicators. It identified Intelligent Smart city as a city with a good future outlook in terms of six aspects, namely smart economy, smart environment, smart living; smart people, smart mobility, and smart governance and another dimension was added which is smart data adopted from [47, 48, 60, 61]. More than 90 indicators covering the six categories were reviewed and in relevance to addressing resilience and targeting urban scale. Only 54 indicators were selected as shown in Fig. 2 categorized according to the selected European approach adopted from the reviewed literature. The indicators are given codes from S01 to S54 to be included in the framework proposed by this study.

## Methods

**Fig. 3:**
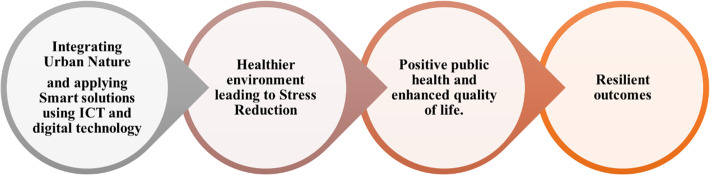
Building a conceptual framework of using smart and biophilic solutions for urban resilience (Authors)

Different researches were conducted to identify indicators for both biophilic and smart cities in addition to different models and frameworks. However, the concept is still inconsistent; hence, appropriate and specific indicators are needed to be developed on different levels to ensure achieving urban resilience through biophilic and smart approaches. Therefore, this research adopts a descriptive-analytical approach in order to identify the interrelationship between principles of urban resilience, biophilic, and smart city indicators. This aims to answer the proposed questions regarding achieving urban resilience through biophilic and smart solutions in contemporary cities, as illustrated in Fig. 3.

It is not a new approach to merge between biophilia and smartness. The idea has been investigated as a solution for compact cities. It has been argued that biophilic approaches are complimentary for smart ones. Moreover, it boosts sustainability and resilient approaches. Melbourne in Australia has given an example for linking both biophilic and smart or digital networks, by giving their trees email addresses linking it to human data networks [63, 64]. This initiative is named “Melbourne Urban Forest Visual”; people can easily visualize the forests’ trees and even send it an e-mail [64].

**Fig. 4:**
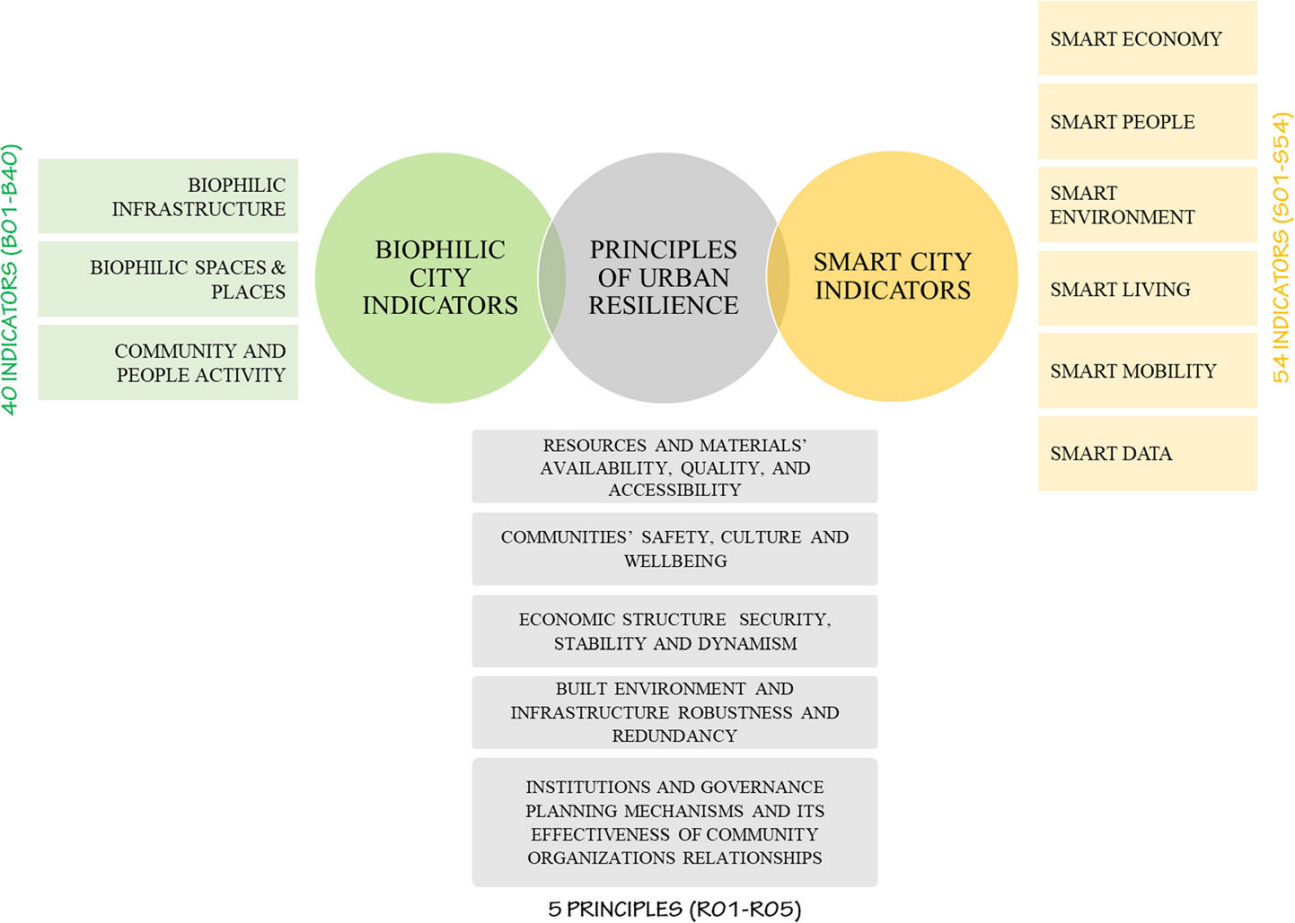
Achieving urban resilience through biophilic and smart approaches (Authors)

Technology can stimulate and enhance biophilic responses via digital and virtual methods and this proves that biophilic and smart approaches might be eventually intersecting; as both approaches can use digital data and target connecting people and nature together. Integration between both aspects could be achieved by dealing with urban biophilia as a distributed system and merging it with digital technology and media will improve its civilizing functions [30, 65]. However, each of the biophilic concepts and smart city ideas was applied separately to achieve resilience without much intersections on the level of indicators as appears in Fig. 4.

The authors ventured to outline the likely cross-relationships between indicators for biophilic cities, indicators for smart cities as discussed in [66–68] with principles for urban resilience at the core. These relationships give a deeper insight into reading urban resilience in terms of balancing between returning back to nature and looking forward to future technologies. It also answers the first addressed research questions about the role biophilic and smart interventions play in attaining urban resilience. The authors had to take every principle of urban resilience and test theoretically its relationship with each biophilic city and smart city indicators. One principle at a time was researched thoroughly before they were all collated together to present the final relationship diagram of all the resilience principles and the indicators of biophilic cities and smart cities at the end of this section.

**Fig. 5:**
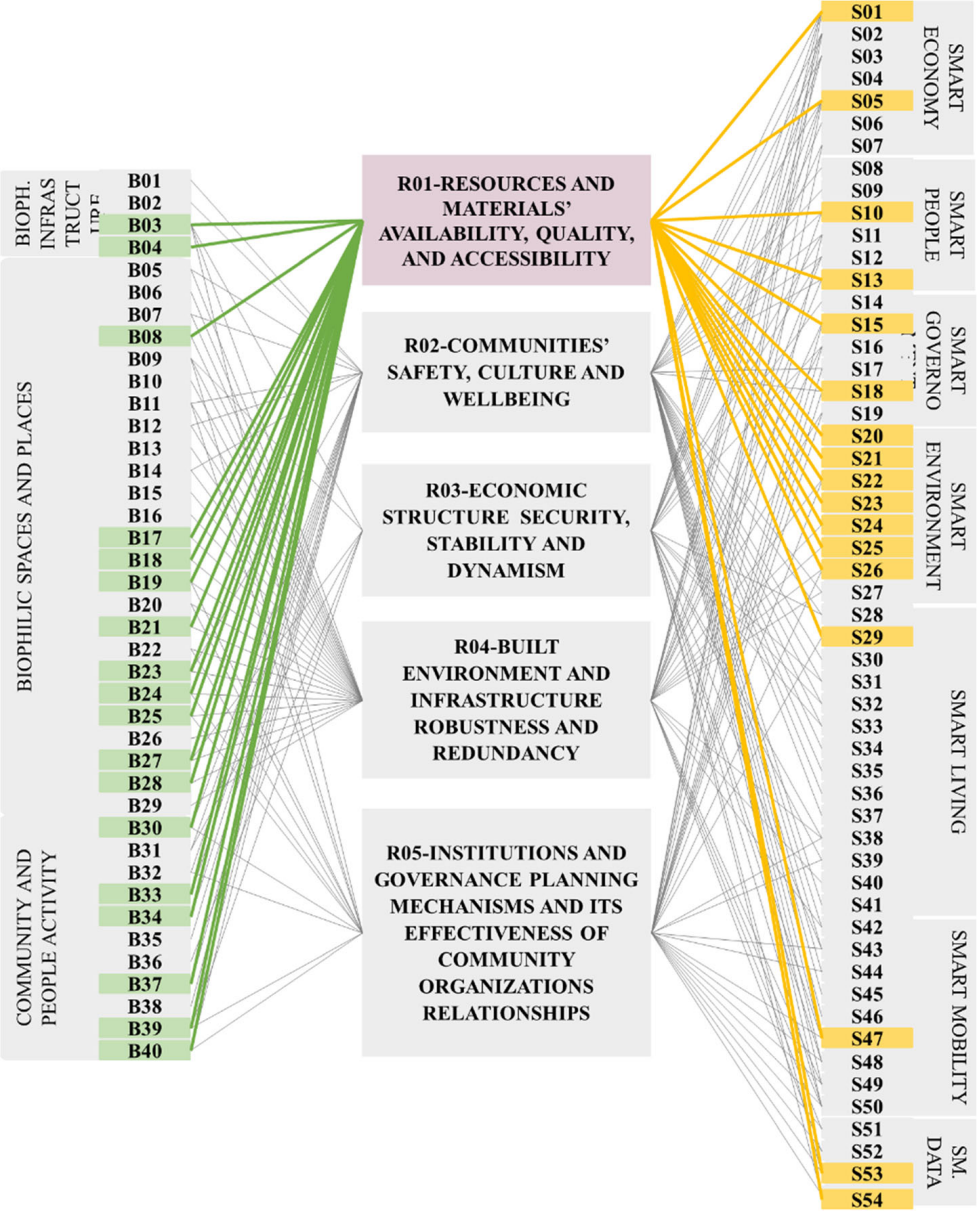
Illustration for the cross-relationships between principles of urban resilience and indicators of biophilic and smart cities (showing R01 as example)

To clarify the authors’ approach to creating proposed relationships between each principle of resilience and biophilic city indicators and smart city indicators, Fig. 5 shows the proposed cross-relationships between (R01) Resources and materials availability, quality, and accessibility, and the relevant indicators of both biophilic cities as an example.

On the biophilic side, it shows the relationship between (R01) and the availability of urban ecological networks (B03) which are presented in natural vegetation, water streams, urban farms, …etc. which presents a strong resource potential that provides essential inputs for production. Similarly, to achieve (R01) Resources availability, quality, and accessibility as a principle of urban resilience, it is essential to consider blue infrastructure (B04), and the area coverage and density of urban trees (B17), vegetated swales (B18), edible landscaping (B19). This will enhance agricultural land and enhance wildlife which in turn will enhance the ecosystem and provide a healthy urban environment [12, 13, 34, 43, 65, 69–74]. Then, all similar relationships between R01 and the rest of the biophilic indicators appear in Table 4 which will not be discussed in detail because it will fog the final objective of this research paper but might be presented in further work.

On the smart city side; the relationship between (R01) *Resources and materials availability, quality, and accessibility*, and research and development expenditure (S01) as an essential indicator for the implementation of smart city strategies and assessment. This is important since it will help in good resources management using different strategies with the help of different digital interventions, in addition to other indicators for smart cities like internet service quality and coverage of broadband (S10) and free Wi-Fi coverage in public spaces (S13) which offers equal opportunities for accessing information and different services (normal and emergencies) in different ways that guarantee efficiency and speed actions. Another example is the significant relation between resources and materials availability and accessibility and smart solid waste collection, disposal, and treatment (S26) which guarantees efficient management for recycling and reusing waste materials in ways that promote zero-waste system achievement [4, 8, 15, 22, 23, 46, 47, 53, 54, 59, 69, 77, 81]. Tables 3 and 4 show further explanation for the cross-relationships between indicators for both approaches and (R01) *Resources and materials availability, quality, and accessibility*. It presents the different sources used to identify and verify the relationship between the indicators of biophilic (Table 3) and smart cities (Table 4) and the principles of urban resilience.

**Table 3:** Illustration for biophilic city indicators and R01 principle of urban resilience

Biophilic city indicators			R01-Resources and materials’ availability, quality, and accessibility	Sources
**Biophilic Infrastructure**	**B03**	**Urban ecological networks**	Presented in natural vegetation, water streams, urban farms, …etc. which presents a strong resource potential that provides essential inputs for production.	[12, 34, 74]
	**B04**	**River systems/floodplains/wetlands**	Considering blue infrastructure is an important resource for achieving resilience by controlling flood and drainage mechanisms with all hydraulic systems to integrate it with agriculture and landscape to increase resources for communities.	[34, 65]
**Biophilic spaces and places**	**B08**	**Daylight interior spaces/dynamic natural light**	Integrating natural light in spaces guarantees direct integration with nature which is essential for health and wellbeing, besides it has a positive impact on energy consumptions for buildings besides reducing overall building energy requirements like heating, cooling, electric lighting)	[34, 65, 82, 83]
	**B17**	**Urban trees**	Integrating urban trees to be a part of ecological strategies since it offers valuable resources for fruits and materials, besides its environmental benefits.	[34, 43, 72, 84]
	**B18**	**Vegetated swales**	Using vegetated swales as sustainable drainage systems for urban areas to enhance resource management and accessibility.	[34, 43, 72, 73]
	**B19**	**Edible landscaping**	Considering green infrastructure will enhance agricultural land and wildlife which in turn will enhance the ecosystem and provide a healthy environment.	[12, 13, 34, 69, 74]
	**B21**	**Stream daylighting, Stream restoration**	Enhancing urban resilience by strengthening the public realm, climate mitigation, and adaptation, through improving public transit, movement modes, and management of rainwater as a resource.	[8, 12, 13, 26, 34]
	**B23**	**Ecology parks**	Protecting park ecosystems as they provide clean air and water in addition to wildlife habitats. It also enables the conservation of natural resources and provides healthy environments.	[11, 15, 26, 38]
	**B24**	**Neighborhood parks and pocket parks**		[12, 13, 34, 69, 74]
	**B25**	**Greening gray fields and brownfields**	Providing safe cleanups and sustainable plans for reusing such areas in cities makes them a good resource for new green spots which enriches cities’ green infrastructure.	[13, 65, 85]
	**B28**	**Regional green space systems**	Conserving green systems is essential for resources management. Planning urban green areas is important for integrating nature into urban settings.	[86, 87]
**Community and people activity**	**B30**	**Urban creeks and riparian areas**	Considering these areas offers a good source for vegetation, green areas, water, and wildlife.	[86, 87]
	**B33**	**City tree canopy**	Prioritizing tree planting in urban areas which increases nature integration in urban settings and provides good urban natural resource management.	[12, 13, 34, 74]
	**B34**	**Community gardens**	Spreading gardens and protecting them represents a good source of clean air, water, and wildlife habitats.	[13, 65, 85, 88]
	**B37**	**Camping grounds**	Offering spaces and areas for residence and services in case of emergencies.	
	**B39**	**Botanic gardens/environmental education initiatives**	Offering good opportunities for protecting plants, trees, and vegetation which supports self-sustaining ecosystems, and provides different plants, food, and material resources.	
	**B40**	**Ecosystem restoration/conservation project**	Integrating social-ecological processes on different scales to manage different resources.	[86, 87]

**Table 4:** Illustration for smart city indicators and R01 principle or urban resilience

Smart city indicators			R01-Resources and materials’ availability, quality, and accessibility	Sources
**Smart economy**	**S01**	**Research and development expenditure**	Applying different innovations and digital interventions in R&D provides good resources management and documentation using different strategies.	[19, 75, 76]
	**S05**	**Number of start-ups**	Increasing start-ups in communities guarantee resources, products, and different services.	[8, 19, 23, 47]
**Smart people**	**S10**	**Internet service quality and coverage of broad bands**	Enhancing internet connections guarantees connectivity and simultaneous access to different resources, in addition to communication requirements.	[15, 21, 46, 77]
	**S13**	**Free Wi-Fi coverage in public spaces**		[8, 23]
	**S15**	**Strong leadership promoting innovation and smart solutions**	Ensuring strong leadership and actions in case of emergencies guarantees prompt decision-making process and good resources management, with the assistance of ICT solutions.	[19, 75, 76]
**Smart governance**	**S18**	**E-governance and online civic engagement in decision making**	Facilitating online public reporting and online citizen participation in the decision-making process. This benefits decisions related to resources allocation and management regarding production and consumption.	[21, 46]
	**S20**	**Sustainable and natural resources management**	Reducing resources consumptions and managing their distribution through purposive economic shifts that promote human capacities.	[8, 78, 79]
**Smart environment**	**S21**	**ICT enabled environment monitoring infrastructure and activities**	Managing different resources and using ICT to guarantee availability, accessibility, and productivity.	[8, 23, 47]
	**S22**	**Environmental ecosystem protection**	Improving the accessibility of environmental information and enhancing environmental conservation. By empowering citizens’ participation in decision-making regarding environmental issues.	[8, 19, 23]
	**S23**	**Energy-efficient management and usage using ICT**	Providing a quantitative basis for controlling resource consumption and reduction besides promoting the efficient use of different resources. Also, ICT could be promoting guidance in water quality management systems which contributes to risk mitigation.	[78, 79]
	**S24**	**Quality monitoring of water**		[19, 75, 76]
	**S25**	**Efficient generation, distribution, and usage of water**		[19, 75, 76, 80]
	**S26**	**Smart solid waste collection, disposal, and treatment**	Creating systems for smart waste disposals and adoption of treatment and recycling technologies that provide different resources like biogas and landfill gas.	[81]
**Smart living**	**S29**	**Smart accessibility to utilities and services**	Providing quick and smart access to different utilities and services promotes resources management and monitoring which control the supply and demand issues.	[8, 47]
**Smart mobility**	**S47**	**ICT usage in management systems**	Managing mobility using smart interventions mitigates climate change and reduces urban noise levels which creates more efficient and healthier environments.	[8, 78, 79]
**Smart data**	**S53**	**Open data platforms**	Improving data accessibility, transparency, and accountability which facilitates different participations and practices in resource’ management.	[47]
	**S54**	**Data availability in open format**		[8, 47]

Moreover, Table 5 and Table 6 were established to trace and support the collated relationships between the five principles of urban resilience (R01–R05) and both biophilic and smart city indicators.

**Table 5:** Mapping relationship between biophilic cities indicators and principles of urban resilience (Authors)

Biophilic city indicators			Urban Resilience principles				
			R01	R02	R03	R04	R05
**Biophilic Infrastructure**		**B01**		√			
		**B02**		√			
		**B03**	√		√	√	√
		**B04**	√				
**Biophilic spaces and places**	**Buildings**	**B05**		√		√	
		**B06**				√	
		**B07**		√		√	
		**B08**	√			√	
		**B09**			√	√	√
		**B10**				√	
		**B11**		√		√	√
	**Block**	**B12**		√		√	
		**B13**				√	
		**B14**		√		√	
	**Street**	**B15**				√	
		**B16**				√	
		**B17**	√			√	
		**B18**	√			√	
		**B19**	√			√	√
		**B20**				√	√
	**Neighbourhood**	**B21**	√			√	
		**B22**				√	√
		**B23**	√	√		√	
		**B24**	√			√	√
		**B25**	√			√	√
	**Region**	**B26**				√	
		**B27**	√		√	√	
		**B28**	√			√	
		**B29**				√	
**Community and people activity**		**B30**	√	√			√
		**B31**		√	√		
		**B32**		√			√
		**B33**	√	√			
		**B34**	√	√			
		**B35**		√			
		**B36**		√	√		
		**B37**	√	√			
		**B38**		√			
		**B39**	√	√	√		√
		**B40**	√	√			√

Legend √ The cross-relation between the indicator and the principles

**Table 6:** Mapping relationship between smart cities indicators and principles of urban resilience (Authors)

Smart city indicators		Urban Resilience principles				
		R01	R02	R03	R04	R05
**Smart Economy**	**S01**	√	√	√	√	√
	**S02**		√	√	√	√
	**S03**		√	√	√	
	**S04**			√		
	**S05**	√	√	√	√	√
	**S06**			√		
	**S07**			√		√
**Smart People**	**S08**		√			
	**S09**		√			
	**S10**	√	√		√	
	**S11**		√	√		√
	**S12**		√	√	√	
	**S13**	√	√		√	
**Smart Governance**	**S14**			√		√
	**S15**	√			√	√
	**S16**			√	√	√
	**S17**		√			√
	**S18**	√	√			√
	**S19**				√	√
**Smart Environment**	**S20**	√				
	**S21**	√				
	**S22**	√			√	√
	**S23**	√				
	**S24**	√			√	
	**S25**	√			√	
	**S26**	√			√	√
	**S27**		√		√	
**Smart Living**	**S28**		√	√		
	**S29**	√				√
	**S30**		√			
	**S31**		√	√		
	**S32**		√	√		
	**S33**		√	√		
	**S34**		√			
	**S35**		√			
	**S36**		√			
	**S37**		√			
	**S38**			√	√	√
	**S39**			√	√	√
	**S40**				√	
	**S41**		√			
**Smart Mobility**	**S42**		√		√	
	**S43**		√	√		√
	**S44**		√			√
	**S45**		√		√	
	**S46**		√		√	
	**S47**	√	√	√		√
	**S48**		√	√		√
	**S49**		√		√	√
	**S50**		√	√	√	√
**Smart Data**	**S51**				√	`√
	**S52**				√	√
	**S53**	√		√		√
	**S54**	√		√		√

**Fig. 6:**
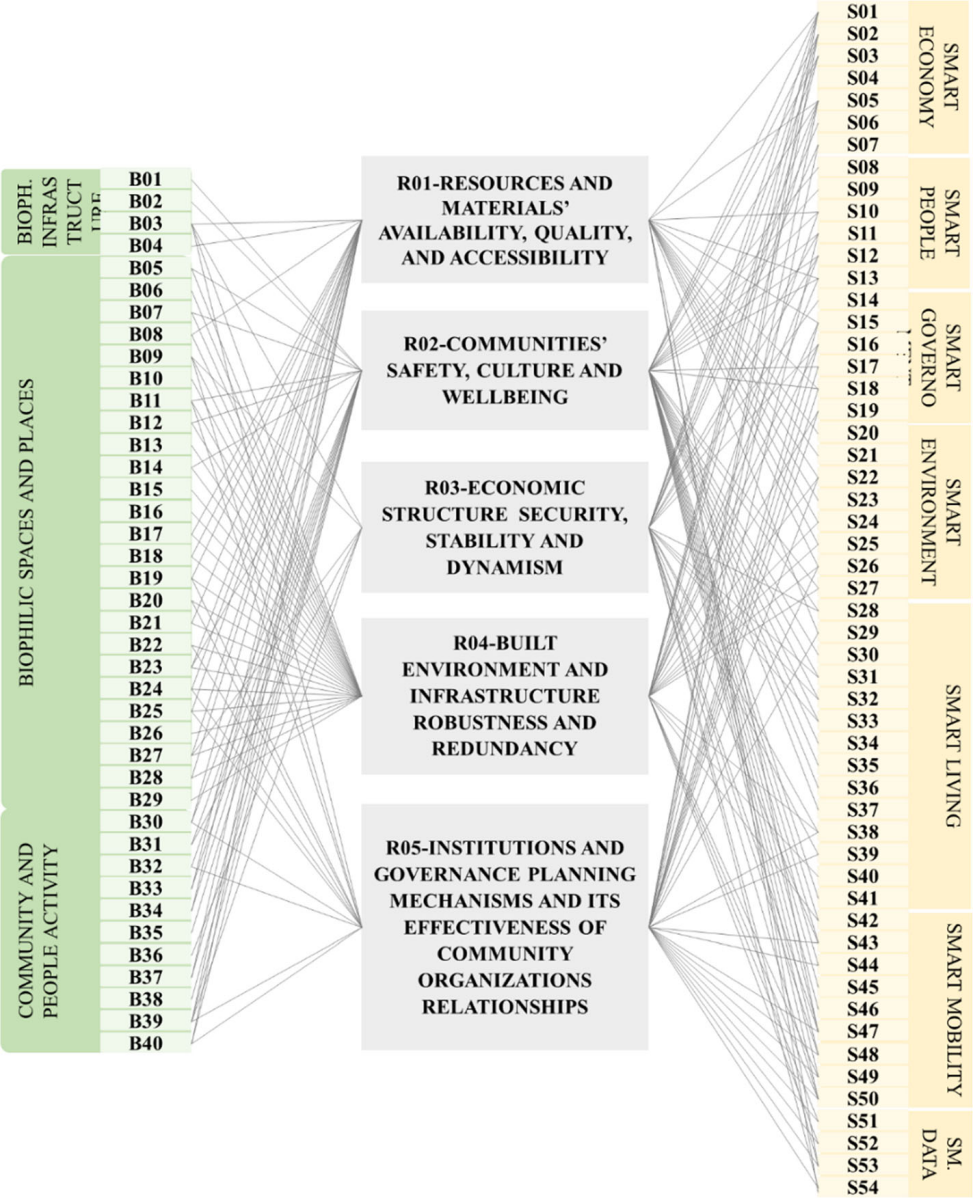
Cross-relationship between principles of urban resilience and indicators of biophilic and smart cities (Authors)

Accordingly, Fig. 6 shows the cross-relationships between the principles of urban resilience and the indicators of both smart and biophilic cities reflecting the authors’ opinion. The proposed relationships (as shown in the diagram) will be the base for more research on the development of a framework for achieving urban resilience through biophilic and smart approaches. It could be regarded as a preliminary guideline for implementing novel biophilic/smart solutions for more resilient cities.

## Results and discussion

Based on the previous assessment, a framework illustrating the main contribution to this study in which the research question was addressed—what role could biophilic and smart cities play in enhancing urban resilience practices?—The idea of the proposed framework is based on Tim Beatley and Peter Newman description for the pathways to urban resilience using biophilic solutions to achieve healthy and effective adaptive capacities leading to resilient outcomes [34] and Papa et. al. (2015) who illustrated different strategies linking resilience and smart city concepts which is very helpful for modeling the idea of a smart resilient city [89]. In addition to the framework developed by Tabiban and Movadeh, which describes five entities to achieve urban resilience (social, economic, environmental, infrastructure and institutional) [90].

**Fig. 7:**
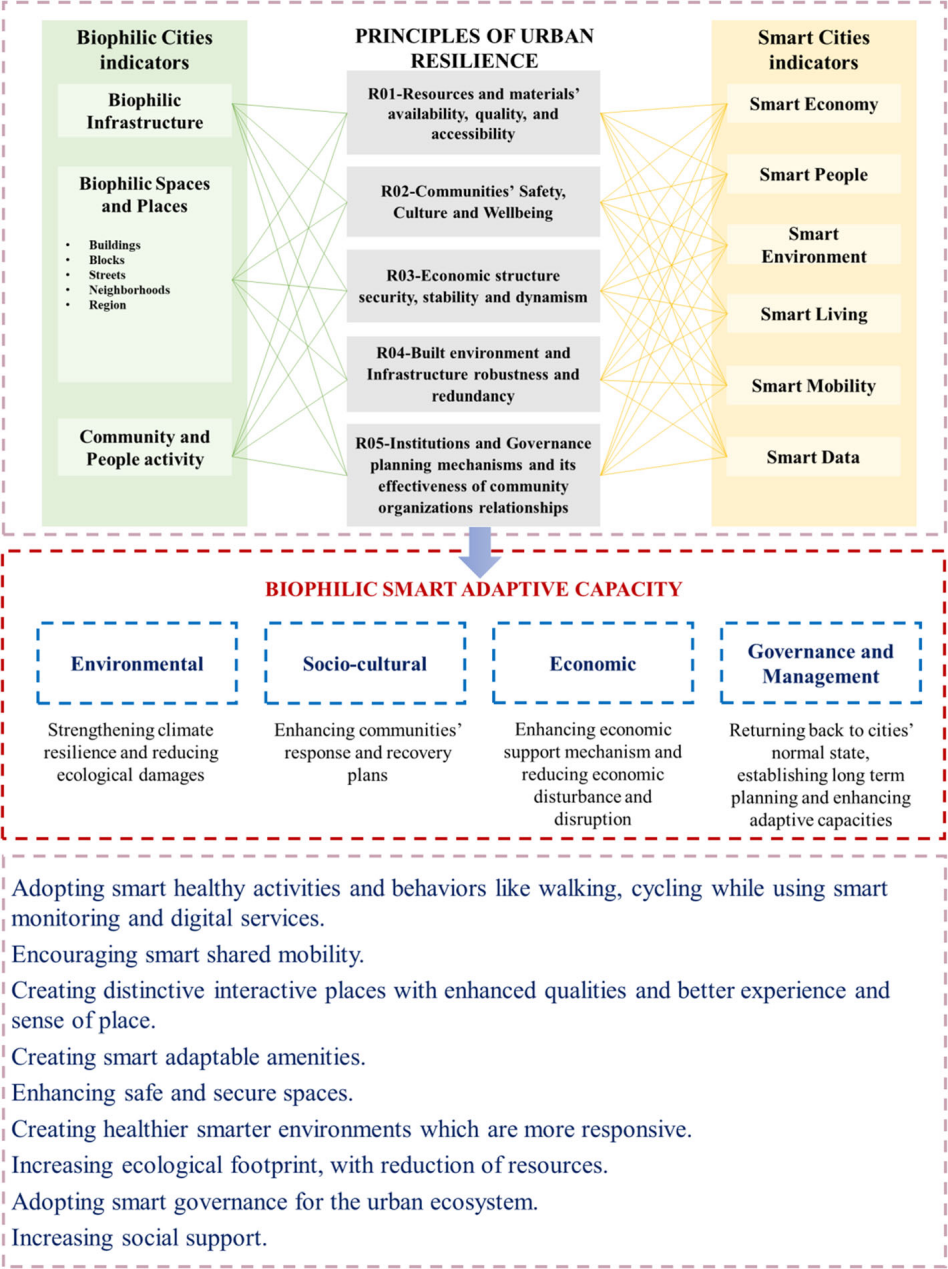
Framework for adopting biophilic and smart cities indicators to achieve urban resilience (as a complement to Fig. 6)

Evidence of the study results proposes implementing smart biophilic approaches, with all its aspects (*Biophilic infrastructure, Biophilic spaces and places and community and people activity*), along with smart city indicators including all its aspects (*smart economy, smart people, smart environment, smart living, smart mobility, and smart data*), all together to fulfill the principles of urban resilience. This aims to reach a biophilic smart adaptive capacity to face the consequences of different disasters and crises. The achieved adaptive capacity could be identified by four dimensions (environmental, socio-cultural, economic and governance, and management) as shown in detail in Fig. 7.

The study illustrates how achieving urban resilience could be promoted depending on nature-based solutions and the adoption of smart solutions, through biophilic and smart approaches. It highlights the essential integration between different systems, methods, and strategies to enhance the integration of nature within contemporary urban settings. Along with the integration of smart ICT solutions supporting communities, the economy, and collaborative governance. In addition to focusing on security, privacy, financial support, and information consistency for the rising requirements for technological updates. In this regard, the proposed framework includes three main phases. The first phase illustrates the integration between principles of urban resilience with indicators of both biophilic cities and smart cities. The second phase is the result of the first one; it shows biophilic smart adaptive capacity. The resulted outcomes are presented in four dimensions: **environmental** which targets reducing ecological damages and increasing climate resilience, **socio-cultural** which targets enhancing responses and recovery plans of communities, **economic** which targets enhancing economic support and reducing economic disturbance and disruptions, and **governance and management** which targets returning cities back to its normal state. All this aims at creating healthier and smarter environments which includes distinctive urban settings and places with enhanced urban qualities that promote people’s urban life. The third phase is the main resilient outcome of the proposed framework; it is presented in general actions representing both biophilic and smart interventions.

These actions fulfill the previously illustrated targets from the second phase, like reducing ecological damages by increasing the ecological footprints and adopting smart governance for urban ecosystems in addition to applying ecosystem restoration and conservation projects. Also enhancing communities’ responses by increasing social support, creating distinctive interactive places, spreading community gardens and pocket parks to enhance people’s connection with nature, and improving community engagement through ICT platforms. Enhancing economic support and reducing economic disruptions by controlling the economic shocks and ensuring the balance between public supply and demand, promoting smart green economy, and expanding urban economic structure. Returning back to cities’ normal state by establishing long-term plans to enhance the adaptive capacities; establishing green space decision-making processes, strategies, and management plans; and developing smart and innovative methods to ensure the completeness of education on different levels, ensuring the gain of knowledge and developing skills of younger generations is essential for community development.

The relationship between principles of urban resilience and both biophilic and smart approaches is reflected in the three phases of the proposed framework. This shows the importance of updating a long-term vision for more integration of biophilic and smart interventions in urban design and planning strategies. This enhances the redundancy of communities, buildings, and infrastructure.

It helps in increasing public health by creating a safer and healthier natural urban environment that connects people more with nature.

The various facets of the highlighted empirical study supported the notion that “implementing biophilic and smart strategies through the integration of different urban resilience dimensions is essential to achieve healthier and greener cities to help to face the consequences of different disasters” and answers the second proposed research question - will adopting biophilic and smart approaches provide new solutions and strategies for contemporary urban areas?—which is also highlighted the integration between different systems and processes for enhancing the role of smart and biophilic approaches supporting the economy and governance, besides socio-cultural and economic aspects.

## Conclusions

This study identified the likely indicators for biophilic and smart cities that could be adopted together in a way to achieve cities’ resilience. The research study identified the deployed key indicators for both biophilic and smart cities. This occurred by reviewing previous researches, case studies, and initiatives regarding biophilic cities and smart cities. This study also recalled the main principles and dimensions of urban resilience to understand how it works and to point out its interrelationship with biophilic smart approaches.

The research study synthesized the indicators of smart and biophilic indicators with the reviewed urban resilience principles. It points out in the light of achieving urban resilience; decision-makers should focus more on smart and biophilic solutions that might have substantial contributions relevant to urban resilience. These solutions should be responding to various pressures and changes in environmental conditions, socio-economic needs and enhancing safety and health for communities. Findings answered the research questions regarding the role that biophilic and smart approaches could play in achieving urban resilience. Disasters alter how cities are governed and administered. In light of this; the presented study proposes new natural and smart interventions. Both biophilic and smart interventions could enhance the functionality of cities’ systems and components, with the aid of research and development, adopting green strategies to achieve a healthier environment and strong adaptive city structure in face of disasters. This could be enhanced by adopting smart and new digital solutions to offer different solutions for various situations in contemporary urban areas, which specifically need new updated tools.

Results show a significant contribution of biophilic and smart interventions that encourages cities to become more resilient which complies with Russo and Cirella who discussed the potential of biophilic smart cities and its benefits to eco-system services and contemporary urbanization [30] and in ecological applications as discussed in [1]. Moreover, comparing the research results to the recent studies in the field, it must be pointed out that the proposed biophilic-smart adaptive capacities to achieve urban resilience can help in promoting smart and sustainable urban development as suggested by Abusaada and Elshater since it contributes to the distinctiveness in smart cities [91].

The research study has some limitations. The lack of prior data combining both biophilic and smart city indicators. Reviewing and analyzing biophilic and smart literature showed that there are important gaps in this field of research. This resulted in subjectivity in the selection of the relevant indicators. Besides, there is a lack of the existence of comprehensive frameworks and methodologies that could be used as tools for evaluating concepts of biophilia and smart city dimensions contributing to urban resilience. Therefore, integration between both biophilic and smart interventions must be investigated deeply.

Findings and conclusions point out suggestions for future research, namely further development of the proposed framework that incorporates biophilic and smart cities’ indicators and urban resilience principles through a quantitative approach and further analysis of previous case studies, in addition to developing a decision-making method or tool to achieve resilient strategies using biophilic and smart city indicators altogether and mapping its contribution to UN SDGs to assist in achieving sustainability for communities.

## Data Availability

All data generated or analyzed during this study are included in this published article.

## Abbreviations

ICT: Information communication technology
SDGs: Sustainable development goals
